# Experimental study on tourist satisfaction using participatory simulation in a virtual environment

**DOI:** 10.1186/2193-1801-2-552

**Published:** 2013-10-22

**Authors:** Dingding Chao, Taro Kanno, Kazuo Furuta

**Affiliations:** Department of Systems Innovation, Graduate School of Engineering, the University of Tokyo, Hongo 7-3-1, Bunkyo-ku, Tokyo, Japan; Resilience Engineering Research Center, Graduate School of Engineering, the University of Tokyo, Hongo 7-3-1, Bunkyo-ku, Tokyo, Japan

**Keywords:** Consumer behavior and satisfaction, Contrast bias, Computer simulation, Virtual environment, Participatory simulation

## Abstract

Before traveling, tourists need to ensure that they will have a well-organized trip, which mainly involves a smooth flow of visits to different tourist attractions by themselves or following a pre-designed plan made by tourism service providers. The present study examined how the sequence of visiting tourist attractions influences tourist satisfaction at the expectation and experience levels. Participatory simulation with a virtual environment platform was employed in the experiments to assess their experience. The research reveals that the contrast bias has significant influence on the assessment.

**Electronic supplementary material:**

The online version of this article (doi:10.1186/2193-1801-2-552) contains supplementary material, which is available to authorized users.

## Introduction

Tourist satisfaction is one of the most important indicators of the future intention of tourists to visit (Osti et al. 2012). Tourism providers can improve their services by studying tourist satisfaction, especially their evaluation processes and preferences. In many studies about tourist satisfaction, the behavior model of satisfaction assessment is derived from studies about the consumers of general commercial products (e.g., Seddighi and Theocharous 2002; Tussyadiah 2006), rather than services that involve abundant physiological fluctuation and complex experience. Text-based questionnaires without further emotional interaction are commonly used to collect data for analyzing the assessment of tourists of their experience. However, human preferences are not constant and their assessment for satisfaction might not have a very rational or standardized basis as suggested in several studies about psychology and behavioral economics (Ariely and Zauberman 2000; Kahneman 2000a). Thus, tourist satisfaction with their expected or actual experience at each tourism attraction can be influenced largely by different sequences of visiting.

Contrast bias describes the inclination to overrate or underrate a subject compared with another (Herr et al. 1983). The present study adopts these concepts to refer to assessment biases with regard to tourist satisfaction. The contrast bias is exhibited when the sequence of visiting tourist attractions is changed in different contexts (e.g., the types of attraction and perceived quality of the service). In other words, previous travel experiences of tourists affect their assessment of the satisfaction of their next travel experiences.

Our study intends to explore the contrast bias on tourist satisfaction by participatory simulation in virtual environments (VEs). By combining experiments with the participation of real people and artificial tourism scenarios, we examine the theories beyond the simulation settings, thereby deepening our understanding of the assessment of tourists of their experience.

## Previous studies and hypothesis development

### Overview

Some researchers have suggested that tourists assess their expected or experienced satisfaction with destinations through the “characteristics” they possess (Seddighi and Theocharous 2002; Tussyadiah 2006). The researchers argue that tourists “consume” each characteristic (e.g., natural beauty, cultural heritage, and entertainment elements) of the tourism destination. Most studies about tourist behavior were inspired by research about consumer behavior (Lancaster 1966), and other research results in tourism (Wu and Carson 2008; Yang et al. 2009). However, in this assessment model, tourists have very rational and stable preferences towards different types of attractions and destinations, which may contradict reality (e.g., Kahneman and Tversky 1979; Kahneman 2000a).

Studies in behavioral economics revealed that consumers are not only rational as modeled in many other studies, but their preferences also seem to be easily framed and their decisions are reversed under different contexts (Ariely and Zauberman 2000). Interesting findings on behavioral economics, such as affection treadmill (Kahneman 2000b) and hedonic adaptation (Frederick and Loewenstein 1999), provide new perspectives to improve the model for the general assessment of tourists of their experience.

### Hedonic treadmill and perceived variety

The term “hedonic treadmill” was brought up in wellness studies (Diener 2000). “Hedonic treadmill” generally means the phenomenon in which people have a very strong capability to adapt to pleasant or painful events that happen in their life, and thus move back to an emotional baseline prior to the changes (Myers and Diener 1996). Many other researchers have extended the definition by distinguishing different types of “treadmill” according to their adaptation channel (Kahneman 2000b). The present study simply adopted the general concept of human attentiveness to adapt to pleasant or unpleasant experiences.

As pointed out by many researchers, having a variety of activities is one of the most crucial factors for resisting the hedonic treadmill of happiness in life (Lyubomirsky 2005; Chernev 2011). However, simply increasing the variety may not be the ultimate solution to reduce adaptation (Redden and Haws 2012). Researchers in marketing science have pointed out that categorization that influences the perceived variety of consumer products also effectively affects how consumers rate their enjoyment by changing the settings of assertion (Kahn and Wansink 2004). These studies suggested that the differences in the types of context in which the subject is compared with, might also contribute to the contrast bias.

### Related works in tourism studies

Most stakeholders in the tourism industry prefer repeaters, and invest much to attract and nurture loyal visitors, but like many other types of consumers, tourists show a variety-seeking behavior (Kemperman et al. 2000; Jang et al. 2007). Even repeating visitors usually return to the same tourism destination after some time rather than immediately, and the reasons for their return are quite complex (Kozak 2001). Little is known about how the contrast of tourist experience can affect their enjoyment or satisfaction. The present study believes that the difference in the sequence of visiting, which leads to the contrast of different tourist experiences, affects the judgment of tourists regarding their satisfaction. The results of this study will help travel agencies to improve their services, especially for arranging guided tours. The hypothesis is important for tourism-related information providers (websites, publishers) in organizing the information and spotlighting promotions.

## Methodology

### OpenSimulator

Some researchers in tourism services have successfully applied information technology to assist their studies of the assessment of tourists of their experience (Gretzel 2011; Johnson and Sieber 2011; Yang et al. 2009). Information systems are powerful tools in developing decision-making support systems, conducting observation, collecting data, and developing simulations. However, as tourist experience involves abundant physical and emotional interactions between tourists and tourism spaces, the present study needs to develop a thorough and efficient method of studying the behavioral patterns of tourists, especially their dynamic, emotional, and sometimes, irrational decision-making. The recent trend of applying VEs in assessing user experience solves such a problem by providing assistance in creating and controlling interactive and real-world-like environments (Rebelo et al. 2012). Hence, to deepen our understanding of the assessment of tourists of their experience and to examine the contrast bias, the present study proposes the application of VEs, i.e., artificial but real tourism destination-like, and the use of participatory simulations to observe the assessment tourists of their experience.

OpenSimulator was chosen as the platform to construct our VEs because of the following reasons (Fishwick and Henderson 2008):

1. OpenSimulator is an open-source platform, which allows users to create, customize, and access VEs.
2. It supports real-time interactive three-dimensional (3D) applications to create vivid tourism experience for participants.
3. It allows real time recording of the process and the assessment of tourists.

Although most studies that applied this software are not in the area of tourism services, some tourism services have successfully utilized the OpenSimulator or similar VE platforms to develop simulation platforms for education (Moschini 2010; Sheehy 2010) and study of human behavior (Koutsabasis et al. 2012). We believe that OpenSimulator can also be utilized to study tourist decision-making and assessment, and thus improve future tourism services.

### Application to tourism studies

We developed participatory simulation with OpenSimulator (OpenSimulatorulator) to examine the contrast bias on tourist satisfaction of their travel experience. The process by which a tourist assesses his/her satisfaction with any kind of tourism experience can be separated into two different levels, namely, expectation and experience (Bosque and Martín 2008). With the help of some other users and developers (Lindakellie) of OpenSimulator in providing contents to design virtual worlds under a creative common license, we developed two different sets of virtual tourism destinations to test the effects at the expectation and experience level.

**Figure 1:**
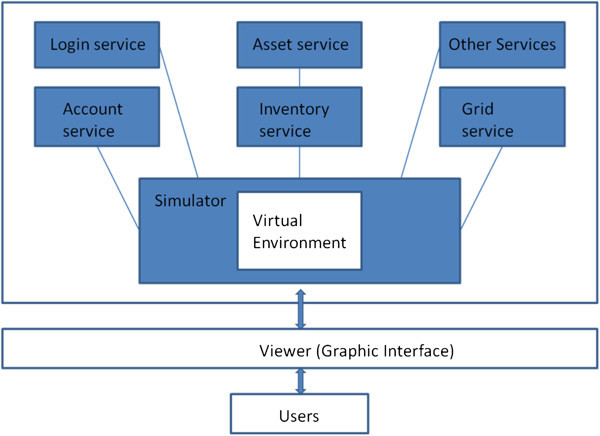
**Structure of the system.**

Figure 1 shows the structure of the system. The system was set up in a stand-alone mode to ensure that the simulator and all services are operating in the same process in only one computer. The contents of VE can be stored as user-inventories and loaded into the simulation scene of the VE. The developer can utilize the “Build” function of the viewer to design and to modify the scene. The users can use the viewer to login to the system, control their avatars, and navigate in the VE.

These are two states of satisfaction during the trip (Oh et al. 2012). The expected satisfaction is usually generated during the decision-making process on destinations or attractions, while experienced satisfaction occurs during travel activities. The expectation level refers to the process where tourists are provided with different options of tourist attractions, and evaluate whether they will be satisfying or not. The experience level refers to the process where tourists evaluate whether tourist attractions were satisfying or not after actually visiting. This study assumes that the contrast bias (of the types of the tourist attraction and the perceived quality of the attraction) can be detected. An attraction varies because of the difference in the visiting sequence, nature of attractions, and expected or experienced satisfaction. The satisfaction may be influenced by the contrast effect of the types of tourist attractions (same or different). However, the contrast bias of the perceived quality of an attraction may result in different experienced satisfaction levels between attractions of high contrast or low contrast of quality pair at the satisfaction level.

### Flow of experiment

#### Pre-experiment session

We obtained 16 screenshots of 16 different tourism destinations/attractions in the VE. The participants were instructed to look at the printed pictures together, and rate them based on the three dimensions of their satisfaction, namely, cultural, natural and entertainment. The participants were then asked to answer their overall subjective assessment of the place. The ratings were all made on a 7-point Likert scale, with scores ranging from 1 (least satisfied) to 7 (most satisfied).

The screenshot of the destinations/attractions that has been used for three formal experiment sessions were also included in the 16 pictures (Additional file 1) to achieve the following:

1. Obtain the tourist original satisfaction assessment
2. Analyze the type and quality of the destinations/attractions to determine whether visiting one after another would be under a high contrast of type frame or a positive/negative contrast of quality frame
3. Compare them with the actual satisfaction assessment under a different frame and bias in the formal session

Data were processed in SPSS by cluster analysis. The semantic distance between each two of the 16 destinations/attractions was calculated based on their average score on the three dimensions (natural, cultural, and entertainment). Next, we defined the destination pairs as “high contrast of type” or “low contrast of type” when the semantic distances between them were significantly large or low, respectively. The average ratings of the assessment of tourists of destinations were also calculated. We defined the destination pairs with significantly different ratings as “high contrast of quality” and others as “low contrast of quality”. In addition, if the better destination was visited before the worse one, the rating of the latter was marked as “negative contrast of quality”. If the better destination was visited after the worse one, the former was marked as “positive contrast of quality”.

#### Experiment 1: frame and bias at the expectation level

**Figure 2:**
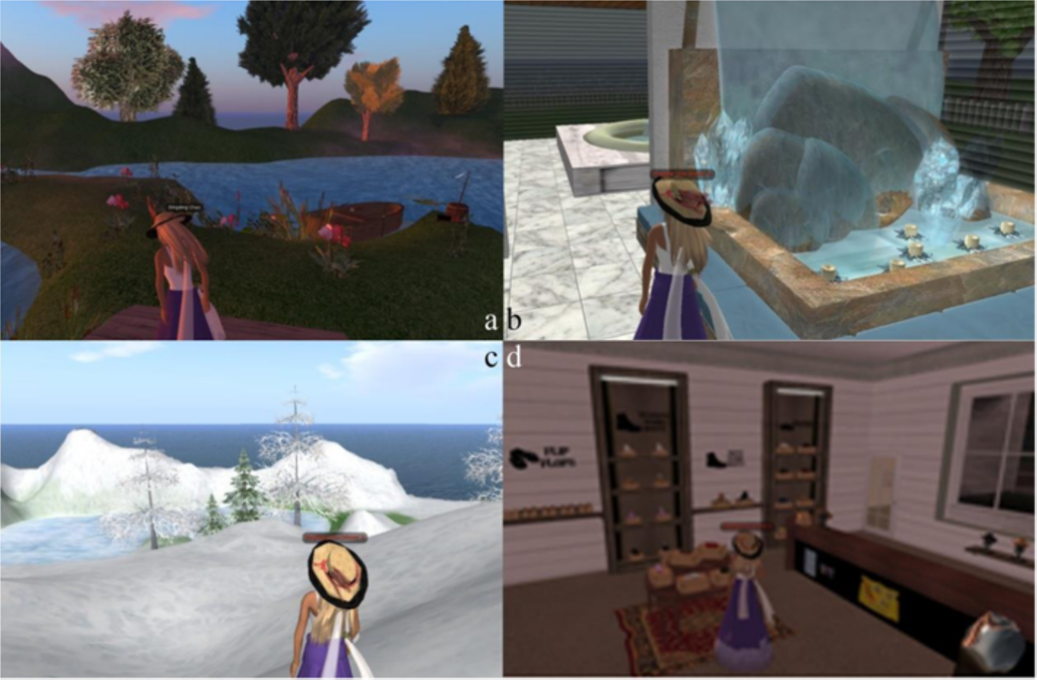
**Screen shots of the virtual attractions at the expectation level: (a) lake, (b) spa, (c) Everest, and (d) shop.**

The VE used for obtaining data at the expectation level has four attractions, namely, lake, spa, Everest, and shop. Snapshots and text related to the attractions were given to the participants to obtain their assessment of the attractions before visiting. Figure 2 shows the screenshots of the virtual attractions: (a) lake, (b) spa, (c) Everest, and (d) shop. Table 1 shows the brief descriptions of the artificial attractions in the simulation environment.

**Table 1:** **Description of the artificial attractions for experiment at the expectation level**

	Brief descriptions of the attractions
Lake	A garden with various types of herbs and a lake
SPA	Spa
Everest	A mountain during winter with a beautiful snow view
Shop	A store in a shopping mall with different brands of shoes

The ratings were made using a 7-point Likert scale, with scores ranging from 1 (least satisfied or unlikely to recommend) to 7 (most satisfied or likely to recommend). The experimental session was conducted as follows:

1. Introductions about four different attractions with pictures and text were individually given to the participants.
2. Once the participants were given the introduction about one attraction, they were given 1 to 2 minutes to read and to imagine their travel experience at the place. They were asked to rate their expected satisfaction (subjective utility) with the attraction.
3. Once all four attractions were rated, the participants were asked to rate the overall expected satisfaction (subjective utility).
4. Finally, the participants were asked to rate their willingness to recommend the whole trip to their friends or family.

Based on the result from the pre-experiment session and the visiting sequence in Experiment 1, we can define the scores that each destination/attraction obtains from the tourists as “high contrast of type” or “low contrast of type”, and “negative contrast of quality” or “positive contrast of quality”.

#### Experiment 2: frame and bias at the experience level

**Figure 3:**
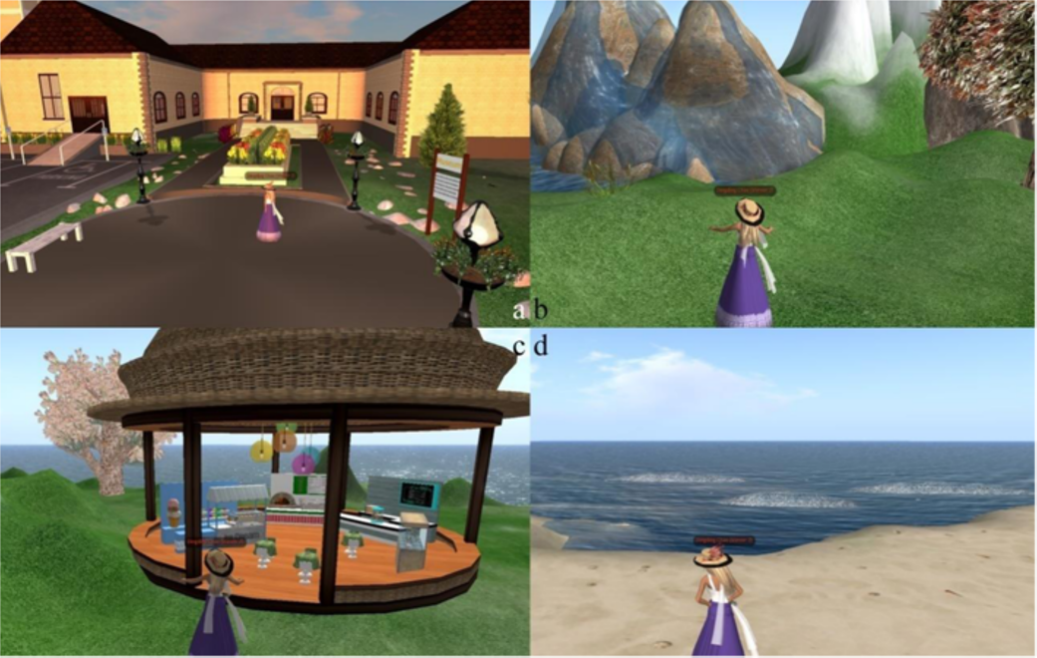
**Screenshots of the virtual attractions at the experience level: (a) gallery, (b) mountain, (c) park, and (d) beach.**

The VE used for obtaining data at the experience level has four attractions, namely, gallery, mountain, park, and beach. We asked participants to tour in the VE by controlling their avatars to walk around the computer-generated 3D world. Figure 3 shows a screenshot of the VE: (a) gallery, (b) mountain, (c) park, and (d) beach. Table 2 presents the brief descriptions of the artificial attractions in the VE.

**Table 2:** **Description of the artificial attractions for the experience level experiment**

	Brief descriptions of the attractions
Gallery	An art gallery filled with paintings and abstract illustrations; Participants can “walk around” and enjoy the art displayed in the gallery
Mountain	A high-rise mountain where participants can climb and enjoy the view along the way
Park	A park with several shops and cable cars
Beach	Seaside where the participants can enjoy a spectacular sunset view

The ratings were made using a 7-point Likert scale, with scores ranging from 1 (least satisfied or unlikely to recommend) to 7 (most satisfied or likely to recommend). The experimental session was conducted as follows:

1. The participants were asked to tour in the VE.
2. Once the participants arrived at an attraction, they had 2 to 3 minutes to walk around the attraction and to rate their satisfaction (subjective utility) of the attraction.
3. Once all four attractions have been rated, the participants were asked to rate the overall satisfaction (subjective utility) of the trip.
4. Finally, the participants were asked to rate how much they were willing to recommend the whole trip to their friends or family.

## Experiment and results

We invited 39 students from the University of Tokyo to participate in the experiment including pre-and after- experiment session. Out of the 39 students, 30 were Mainland Chinese (76.9%), 3 were Taiwanese (7.7%), 4 were Indonesians (10.3%), and 2 were Japanese (5.1%). The participants included 24 males (61.5%) and 15 females (38.5%). Their ages ranged from 20 to 30 (92.3%) years, and only 3 participants were over the age of 30 (7.7%). The experiments were conducted in 6 parts and lasted from 40 to 50 minutes. The participants were asked to participate in the survey to evaluate their satisfaction (subjective utility) as tourists in the virtual tourist attractions. They were paid 1500 yen for their participation. We also asked them to state their personal preferences about the different characteristics of tourism. Most participants reported frequent previous travel experience. For example, 17 out of the 39 participants said that they are very experienced travelers, in or out of Japan (43.6%), while 18 (46.2%) said that they frequently traveled in Japan. The remaining 4 (10.4%) reported that they were not frequent travelers.

Having four destinations/attractions in each experiment resulted in a 4 × 3 × 2 combination, which generated 24 different possibilities of visiting or reviewing sequence in the experiments at both levels. Each participant was assigned to one of the sequences at each level, and each sequence was followed by at least one participant. The participants finished the experiment in 30 to 40 minutes.

### Analysis method and notations

In this study, the contrast bias was confirmed by comparing the satisfaction levels of two attractions that were successively visited. The following two distinctions of successive attractions were considered in the data analysis. However, the attractions at the beginning of visiting sequences were not considered in this analysis because they had no previous attractions.

During the pre-experiment examination, we explored the original preference of the participants and their ratings about the tourism destinations they visited in the formal test. We calculated the semantic distance of the virtual destinations based on their ratings on each characteristic, and grouped them based on their proximity to one another.

Based on the distance between a pair of destinations, we defined Everest-lake, beach-waterfall as the same type of destination/attraction; and shop-Everest, shop-lake, waterfall-gallery, park-gallery, and beach-gallery as different types of destinations/attractions. In the experiments, if the two destinations/attractions that the participants successively visited were of the same type, the rating of the latter was marked as “low contrast of type”. If the successfully visited destinations/ attractions were different, the latter rating was marked as “high contrast of type”. We also defined those destination pairs with significantly different ratings as “high contrast of quality” and others as “low contrast of quality”. In addition, if the better destination was previously visited before the worse one, the rating of the latter was marked as “negative contrast of quality”. If the better destination was visited after the worse one, the former was marked as “positive contrast of quality”.

### Experimental result

#### Contrast bias at the expectation level

The following figures present the results obtained from statistical processing (error bar: 5% of the total scale).

**Figure 4:**
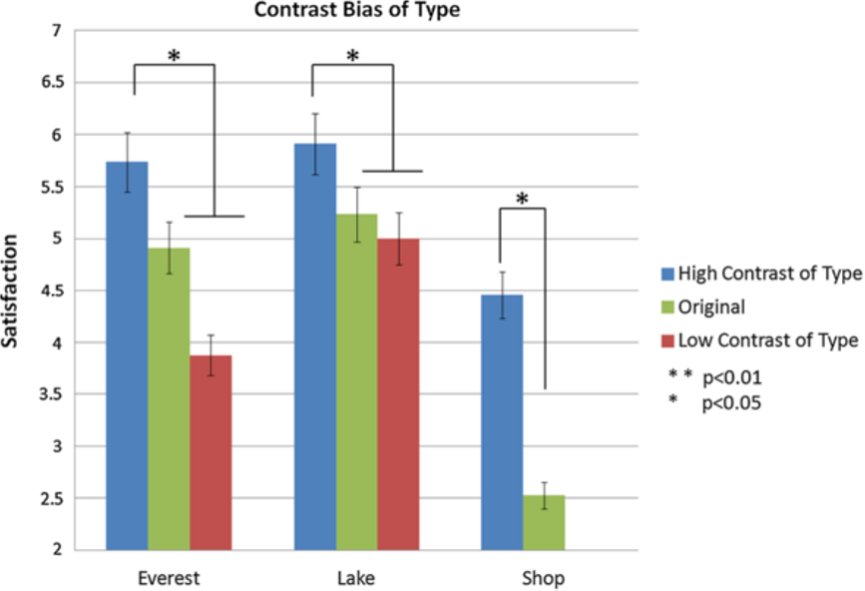
**Contrast bias (types of attraction) at the expectation level.**

Contrast bias is evident in the expectation test (Figure 4). Satisfaction with an attraction is higher when the participants have visited different attractions than when they are visiting the same type. Similarly, satisfaction when under high contrast of type is higher than the original with no contrast. The independent t-test showed the difference is significant between high contrast and original for shop (t=2.42, p<0.05), Everest (t=2.484, p<0.05), and lake (t=1.83, p < 0.05), and between high and low contrast for Everest (t=2.48, p<0.05) and lake (t=2.25, p<0.05). However, the difference between low contrast and original is not significant for Everest (t=−1.319, sig.=0.22) and lake (t=−0.49, sig.=0.63).

For the contrast bias of quality, the effect on satisfaction is not very significant at the expectation level, except for spa and shop. Based on the independent t-test, the difference is significant between negative and positive contrast for shop (t = −2.575, p < 0.05) and spa (t=−3.617, p<0.01).

#### Contrast bias at the experience level

**Figure 5:**
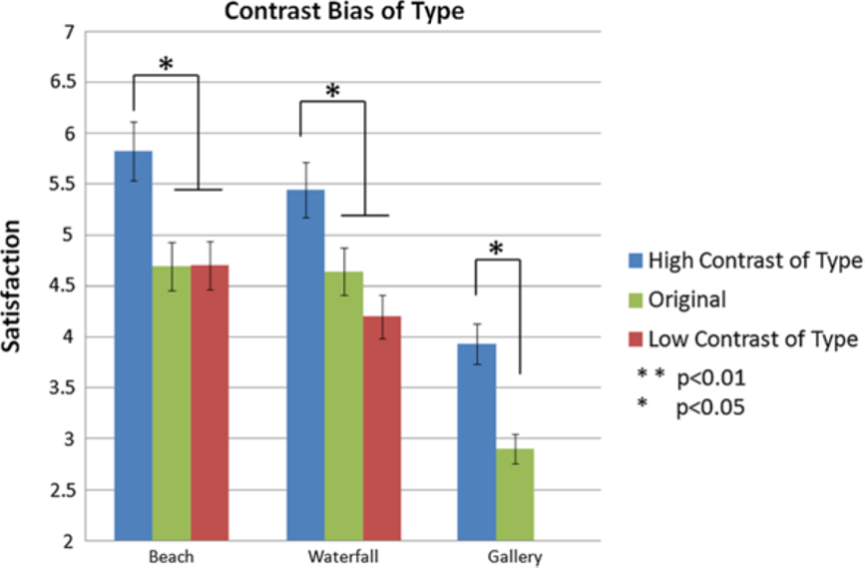
**Contrast bias (types of attraction) at experience level.**

In the experience test, contrast bias on the type of destination shows that satisfaction with an attraction is higher when tourists visit a different attraction than when they visit a similar attraction or the original score. Figure 5 shows the results of the independent t-test in which the difference is significant between high contrast and original for gallery (t=3.003, p<0.05), waterfalls (t=2.320, p<0.05), and beach (t=3.704, p<0.05), and between high and low contrast for waterfalls (t=2.627, p<0.05) and beach (t=2.081, p<0.05). However, the difference between the scores of low contrast and original is not significant.

For the contrast bias of type on information learning at the experience level, the tourists reported high learning about the destination/attraction after they recently visited a different attraction more than after visiting a similar one. The independent t-test showed the difference is significant between high and low contrast for waterfalls (t=1.79, p<0.05) and beach (t=3.01, p<0.01).

**Figure 6:**
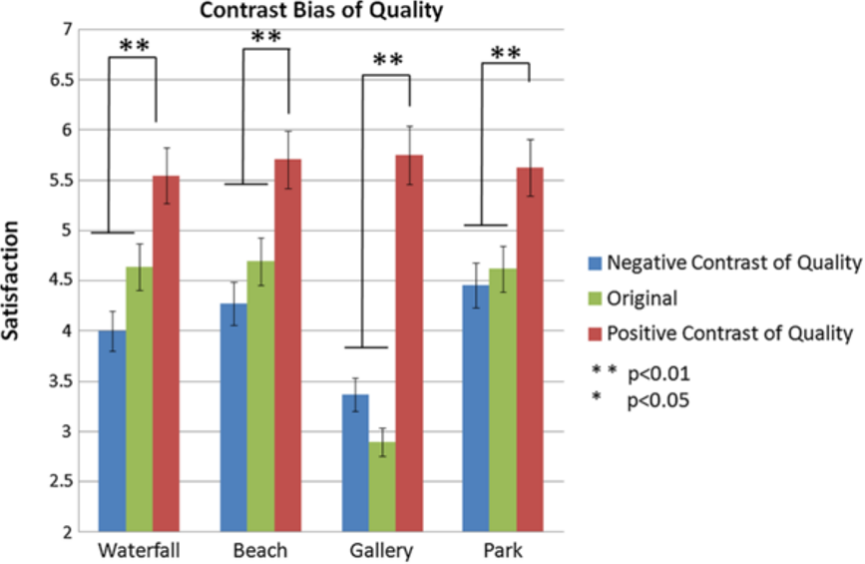
**Contrast bias (quality of attraction) at the experience level.**

A pattern of contrast bias on the quality of attraction implies that satisfaction with an attraction is higher when tourists visit an attraction rated low, and lower when they visit an attraction rated high as shown in Figure 6. Significant is indicated in the results of the independent t-test between negative and positive contrast for gallery (t=−4.431, p<0.01), park (t=−3.167, p < 0.01), waterfalls (t=−3.56, p<0.01), and beach (t=−3.617, p<0.01).

The ability of the data obtained from the VE to reflect the tourist satisfaction assessment in the real world is another key issue in validating the result of the experiment. In the experiment, the participants were requested to evaluate the similarity of their decision/evaluation processes with those they would make in their actual travels. The results are as follows: The average score was 5.35 (SD=1.317) with the minimum score of 3 and the maximum of 7 on the 1 to 7 scale (with 1 indicating “fundamentally different” and 7 indicating “no differences at all”).

The difference between participants’ decision-making in the VE and in reality is marginal and not fundamental.

## Discussions

### Limitations

The results obtained from the experiment reflect the patterns observed in real world data, but the experiment still has the following limitations:

1. The experiment participants are students mainly from Mainland China. Therefore, the results can be bias based on nationality factors.
2. Although in our experiment we confirmed the ability of the data obtained from the VE to reflect the tourist satisfaction assessment in the real world, since the sample is not large, it is not sufficient to prove directly our findings or to validate fully the methodology of using VEs as a platform to study the assessment of tourists of their experience. We propose to perform a small-scale real-world experiment in the future.

The present study applied VEs and participatory simulation to examine the contrast bias in tourist decision-making and assessment. The results of the experiment support our hypothesis that the sequence of visiting, which results in different formation of tourist experience, significantly affects the judgment of tourists on the expected or experienced satisfaction. The results agree with some evidence from real world statistical data.

### Implications

Generally, the contrast bias at the experience level is more apparent than at the expectation level. Moreover, the contrast bias of the perceived quality is more significant than the contrast bias of types at both levels. In previous studies in marketing, neither the expectation vs. experience level nor the contrast bias of perceived quality vs. contrast bias of types has been distinguished, and consumer products rather than tourism services have been considered. This result implies that the sequence of information on attractions provided to tourists influences their expected satisfaction and the organization of the actual trip affects their experience satisfaction with each destination/attraction.

This result will further assist tourism service providers in their decisions regarding marketing and pricing strategy. These providers can then compete well with others, obtain a competitive stance and establish a distinct “brand”. However, changes in the perceived variety seem to have minimal influence on the total satisfaction. This finding suggests that when the service provider intends to promote its tourism products (e.g., guided tour), adding various attractions or destinations alone, which comprise the whole trip cannot ensure increased satisfaction with the entire trip. Moreover, the quality of each attraction should be monitored.

This work showed that participatory simulation through VEs is a promising approach for studying tourist ratings on destinations or attractions. In the future, VE platforms, such as OpenSimulator, should be further utilized for the development of interactive experimental tools to study the assessment of tourists of their experience.

## Electronic supplementary material

Additional file 1: **Appendix A.** Screenshots used for Pre-examination. (PDF 2 MB)

## References

[CR1] Ariely D, Zauberman G (2000). On the making of an experience: the E ects of breaking and combining experiences on their overall evaluation. J Behav Decis Mak.

[CR2] Bosque IR, Martín HS (2008). Tourist satisfaction a cognitive-affective model. Ann Tour Res.

[CR3] Chernev A (2011). Product assortment and consumer choice: an interdisciplinary review. Found Trends® Mark.

[CR4] Diener E (2000). Subjective well-being. The science of happiness and a proposal for a national index. Am Psychol.

[CR5] Fishwick P, Henderson J (2008). Simulating culture: an experiment using a multi-user virtual environment. Proc 2008 winter simul conf.

[CR6] Frederick S, Loewenstein G, Kahneman D, Diener E, Schwarz N (1999). Hedonic adaptation. Wellbeing found hedonic psychol.

[CR7] Gretzel U (2011). Intelligent systems in tourism. Ann Tour Res.

[CR8] Herr PM, Sherman SJ, Fazio RH (1983). On the consequences of priming: assimilation and contrast effects. J Exp Soc Psychol.

[CR9] Jang H, Lee S, Lee S-W, Hong S (2007). Expanding the individual choice-sets model to couples’ honeymoon destination selection process. Tour Manag.

[CR10] Johnson PA, Sieber RE (2011). Negotiating constraints to the adoption of agent-based modeling in tourism planning. Environ Plan B Plan Des.

[CR11] Kahn BE, Wansink B (2004). The influence of assortment structure on perceived variety and consumption quantities. J Consum Res.

[CR12] Kahneman D, Kahneman D, Tversky A (2000). Experienced utility and objective happiness: a moment-based approach, Chapter 37. Choices, Values and Frames.

[CR13] Kahneman D, Kahneman D, Tversky A (2000). Evaluation by moments: past and future, Chapter 38. Choices, Values and Frames.

[CR14] Kahneman D, Tversky A (1979). Prospect theory: an analysis of decision under risk. Econom J Econom Soc.

[CR15] Kemperman ADAM, Borgers AWJ, Oppewal H, Timmermans HJP (2000). Consumer choice of theme parks: a conjoint choice model of seasonality effects and variety seeking behavior. Leis Sci.

[CR16] Koutsabasis P, Vosinakis S, Malisova K, Paparounas N (2012). On the value of virtual worlds for collaborative design. Des Stud.

[CR17] Kozak M (2001). Repeaters’ behavior at two distinct destinations. Ann Tour Res.

[CR18] Lancaster K (1966). A new approach to consumer theory. J Polit Econ.

[CR19] Lindakelliehttp://zadaroo.com/

[CR20] Lyubomirsky S (2005). Pursuing happiness: the architecture of sustainable change. Rev Gen Psychol.

[CR21] Moschini E, Peachey A, Gillen J, Livingstone D, Smith-Robbins S (2010). The second life researcher toolkit – an exploration of inworld tools, methods and approaches for researching educational projects in second life. Res learn virtual worlds SE - 3.

[CR22] Myers DG, Diener E (1996). The pursuit of happiness. Sci Am.

[CR23] Oh J-S, Kim H, Jayakrishnan R (2012). Tourist activity simulation model for assessing real-time tour information systems. J Intell Transp Syst.

[CR24] OpenSimulatorhttp://opensimulator.org/

[CR25] Osti L, Disegna M, Brida JG (2012). Repeat visits and intentions to revisit a sporting event and its nearby destinations. J Vacat Mark.

[CR26] Rebelo F, Noriega P, Duarte E, Soares M (2012). Using virtual reality to assess user experience. Hum Factors J Hum Factors Ergon Soc.

[CR27] Redden JP, Haws KL (2012). Healthy satiation: the role of decreasing desire in effective self-control. J Consum Res.

[CR28] Seddighi HR, Theocharous AL (2002). A model of tourism destination choice: a theoretical and empirical analysis. Tour Manag.

[CR29] Sheehy K, Peachey A, Gillen J, Livingstone D, Smith-Robbins S (2010). Virtual environments: issues and opportunities for researching inclusive educational practices. Res learn virtual worlds SE - 1.

[CR30] Tussyadiah IP (2006). A model of multidestination travel: implications for marketing strategies. J Travel Res.

[CR31] Wu C-L, Carson D (2008). Spatial and temporal tourist dispersal analysis in multiple destination travel. J Travel Res.

[CR32] Yang O, Hui-Min G, Ryan C (2009). Itinerary planning and structured travel—preferences by outbound Chinese holidaymakers. Anatolia.

