# Supplementary figures and images for: Experimental study on tourist satisfaction using participatory simulation in a virtual environment
Source: Springerplus. 2013 Oct 22;2:552. doi: 10.1186/2193-1801-2-552 (PMC4320162; doi:10.1186/2193-1801-2-552)

## Appendix A: Screenshots used for Pre-examination

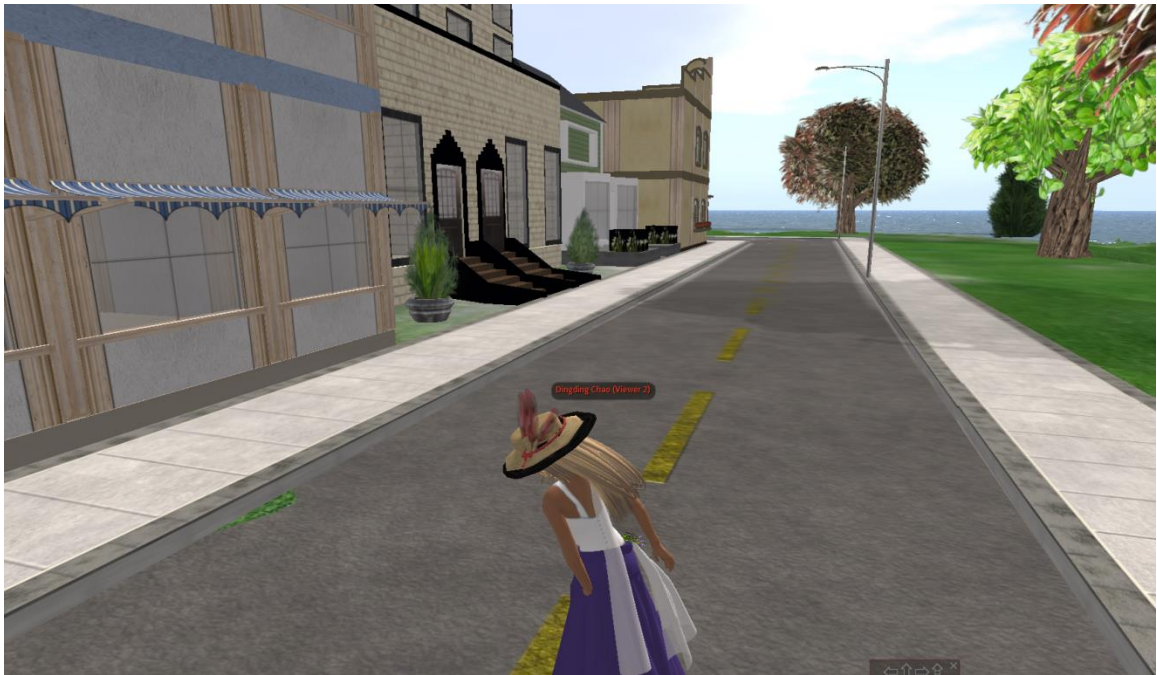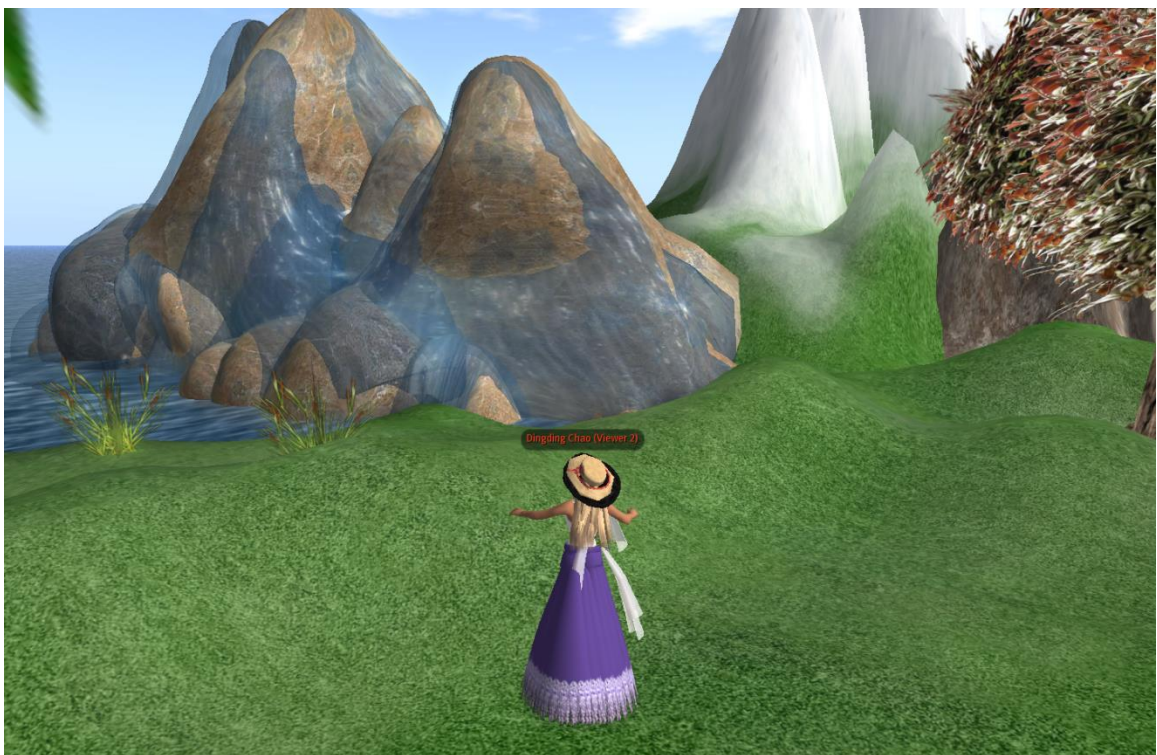

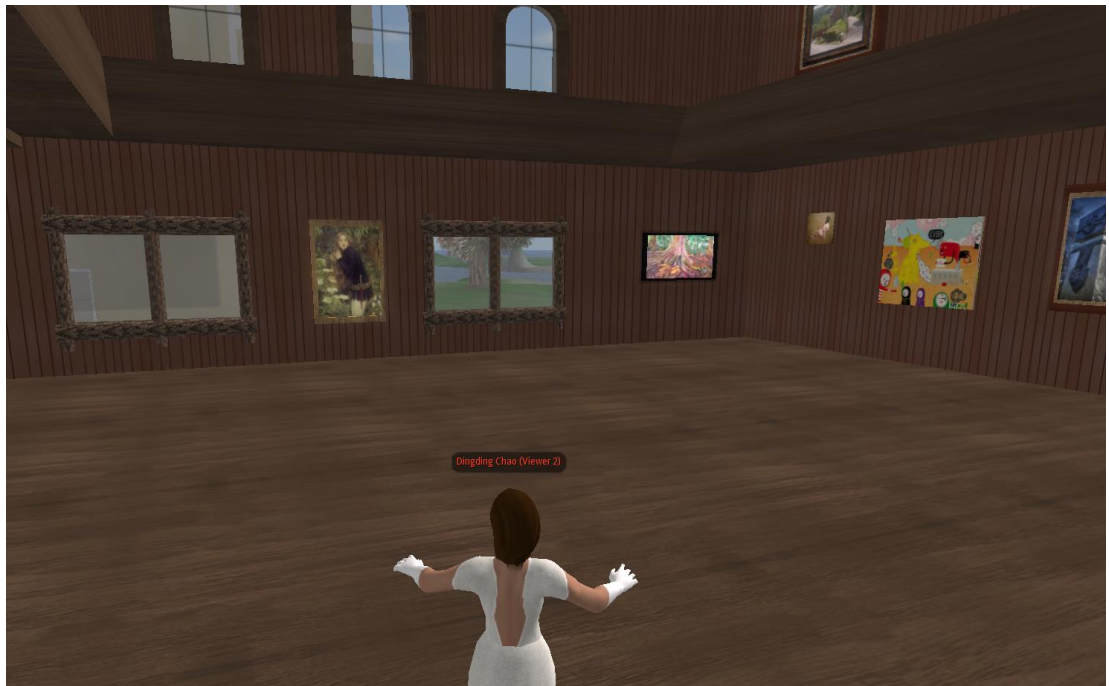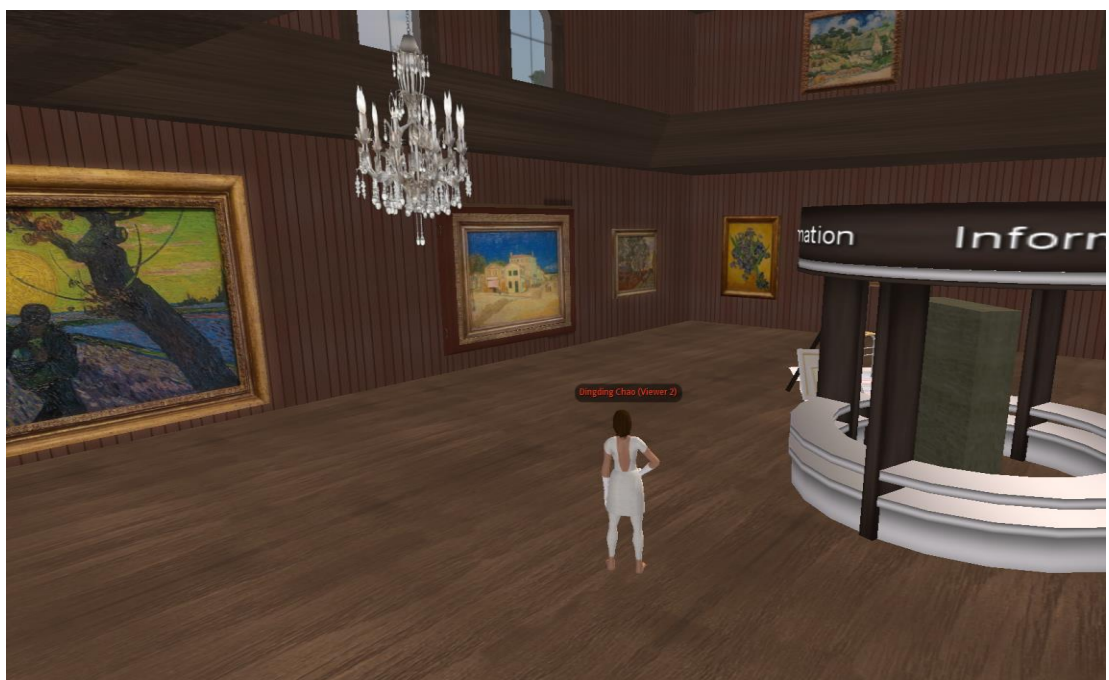

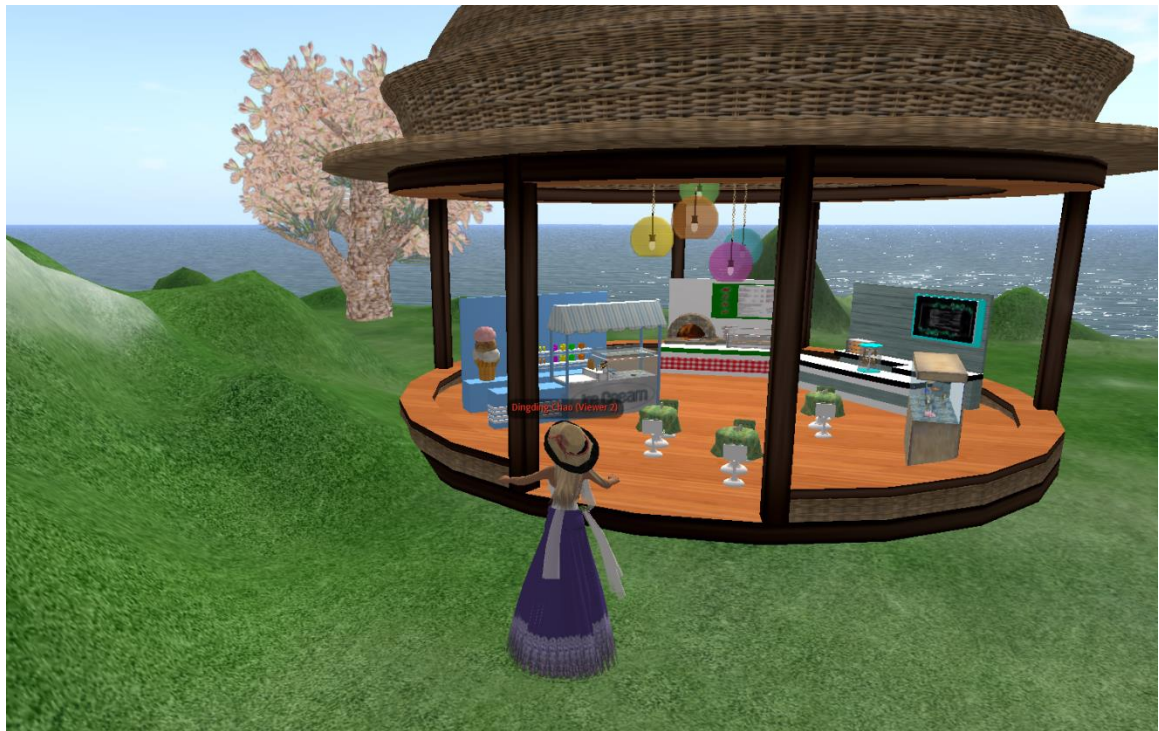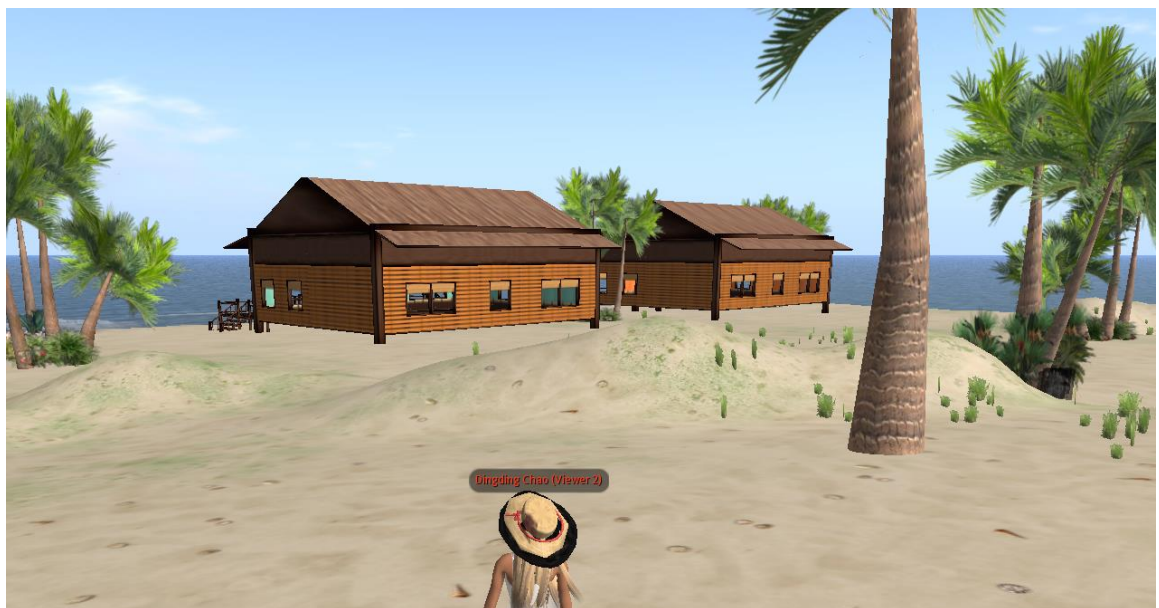

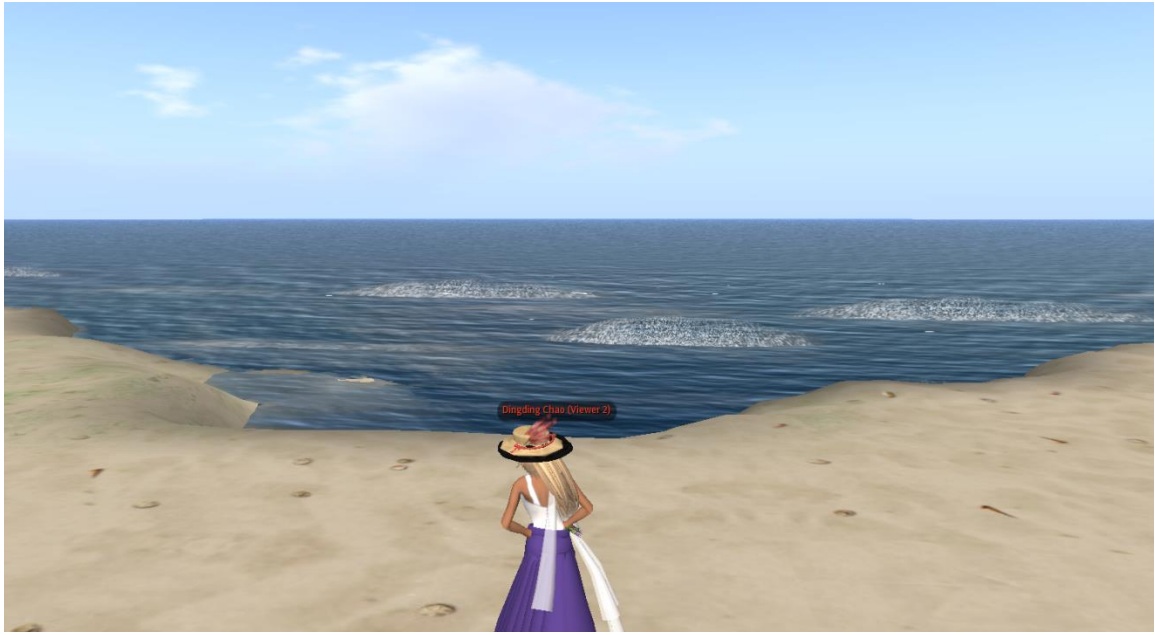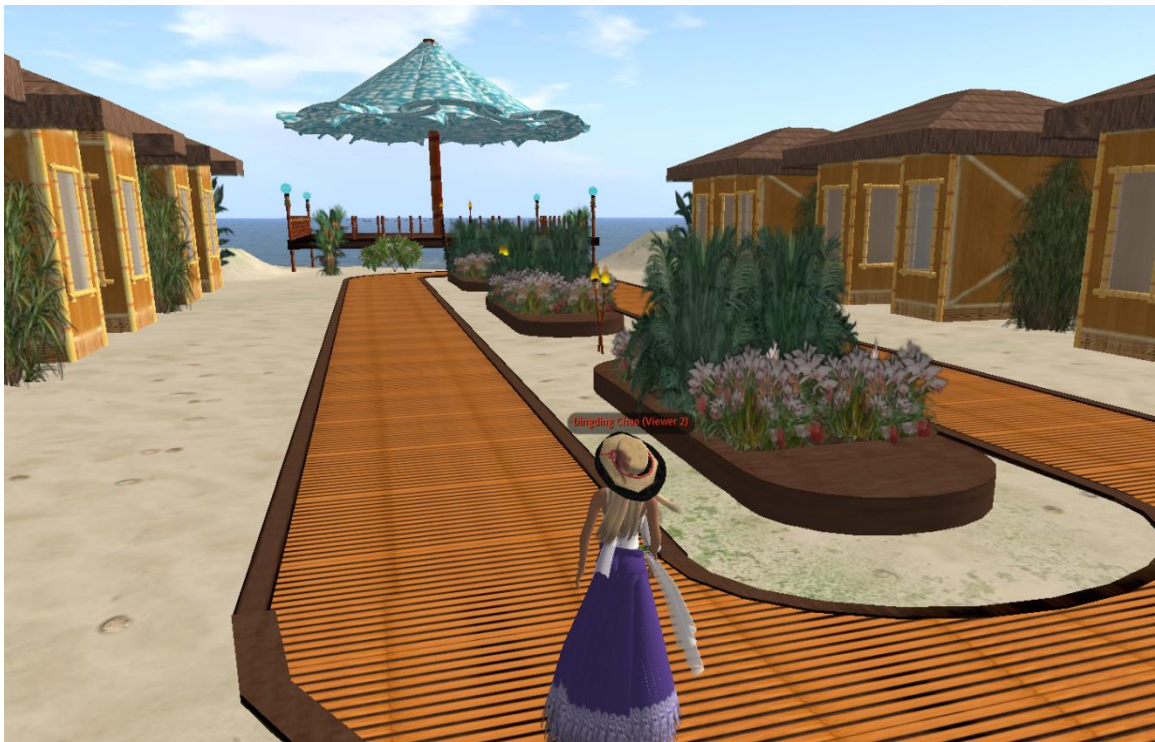

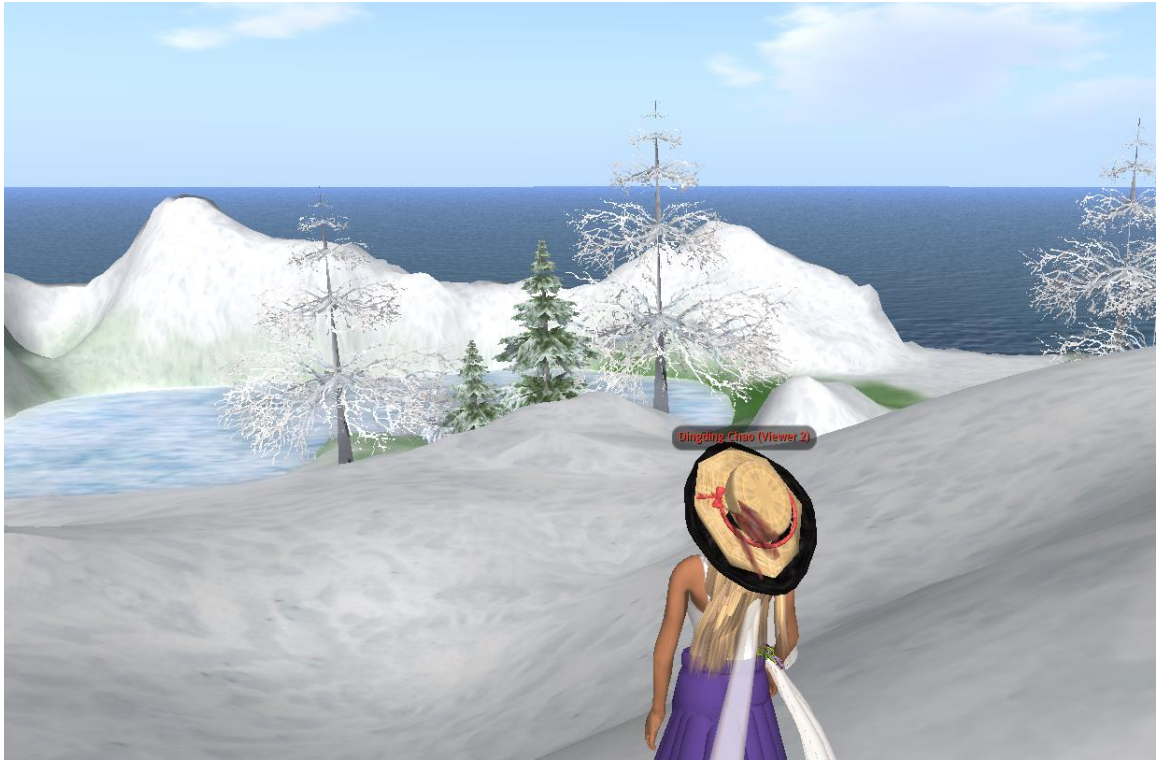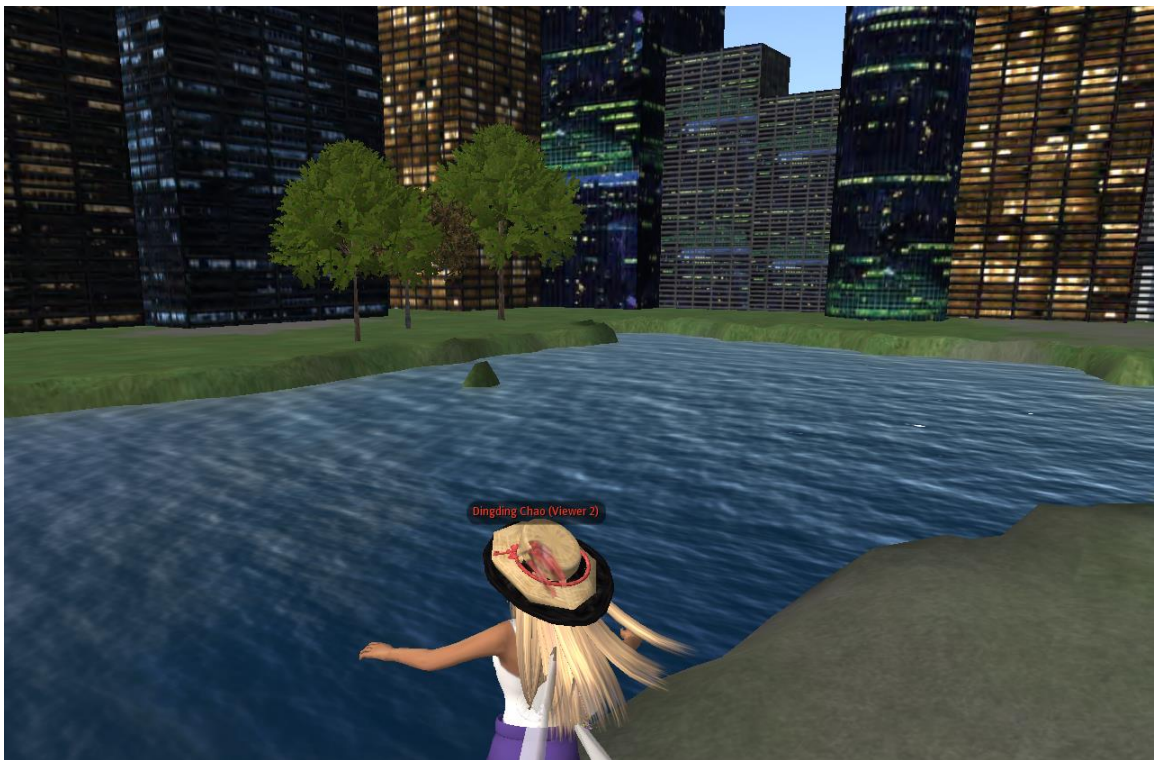

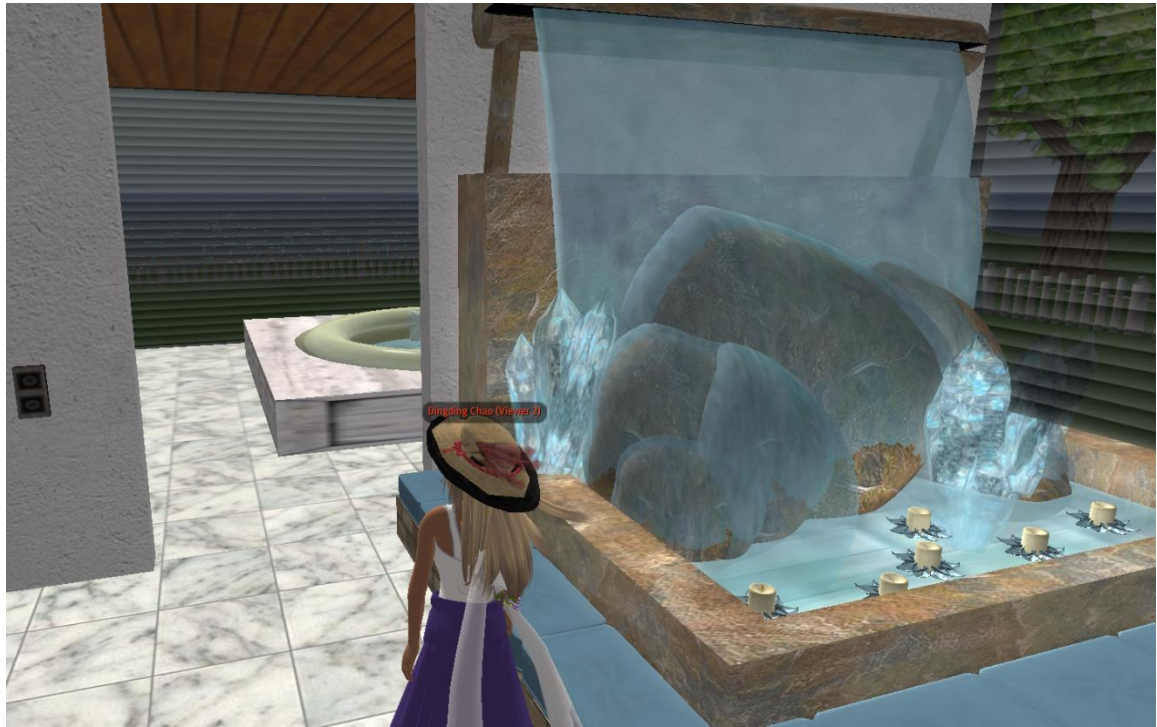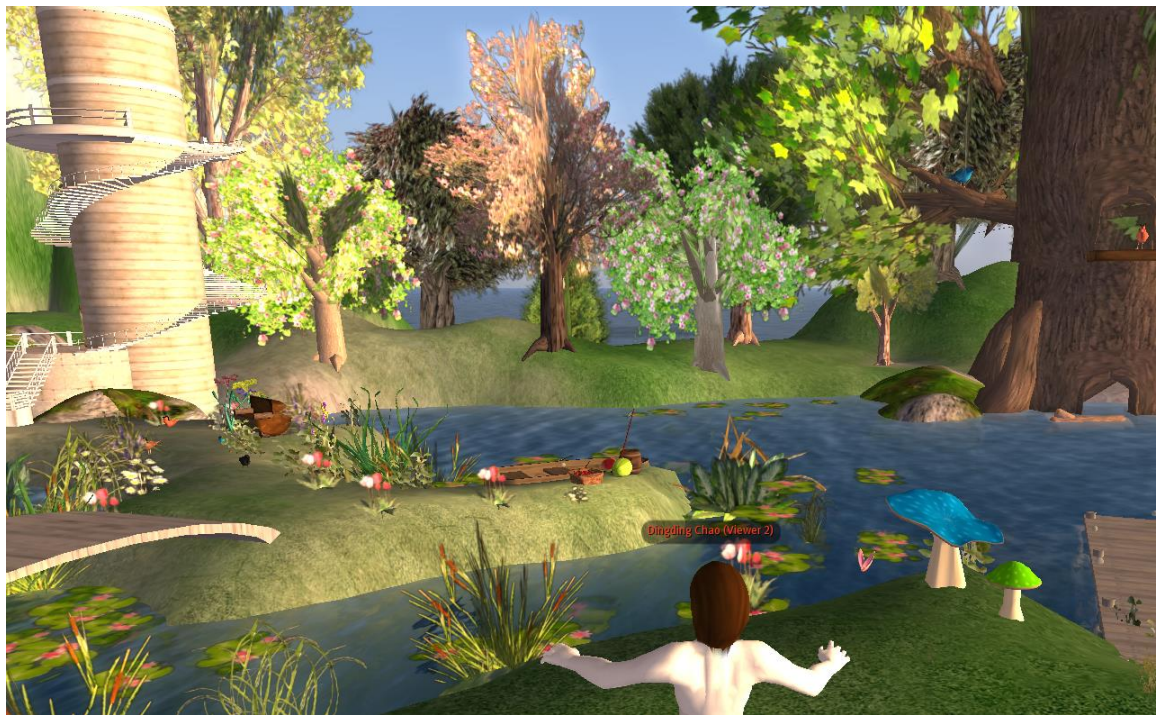

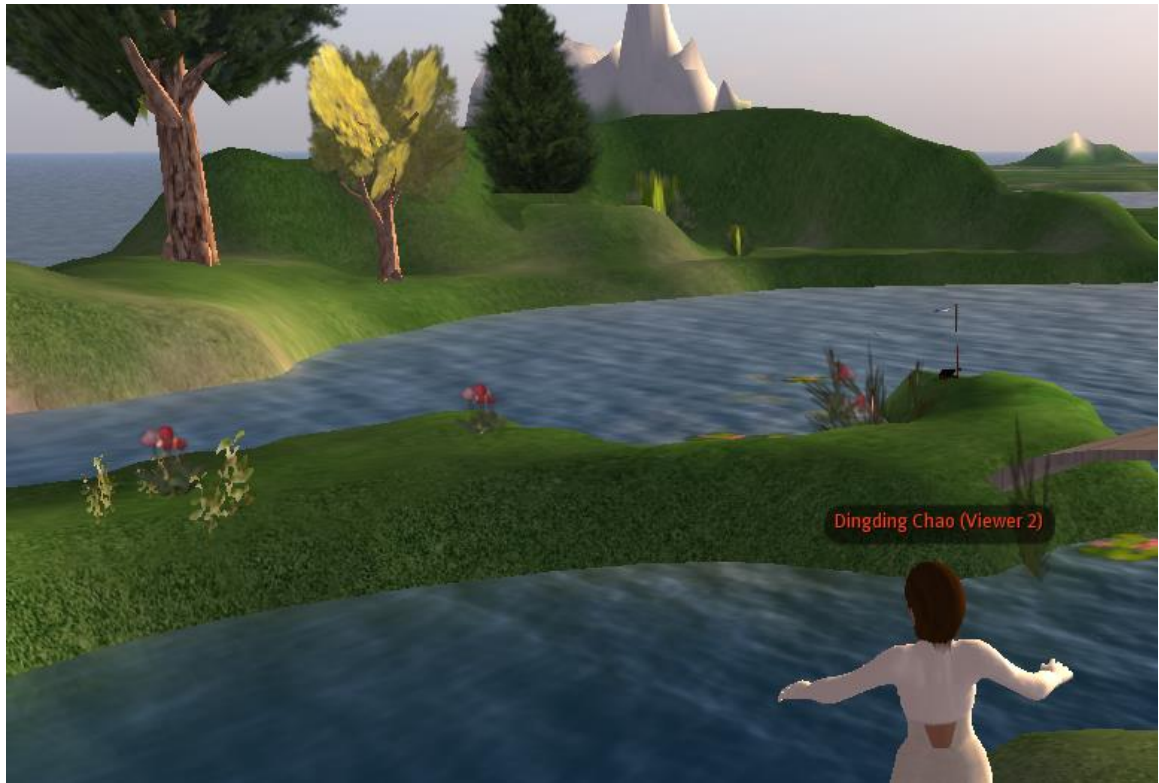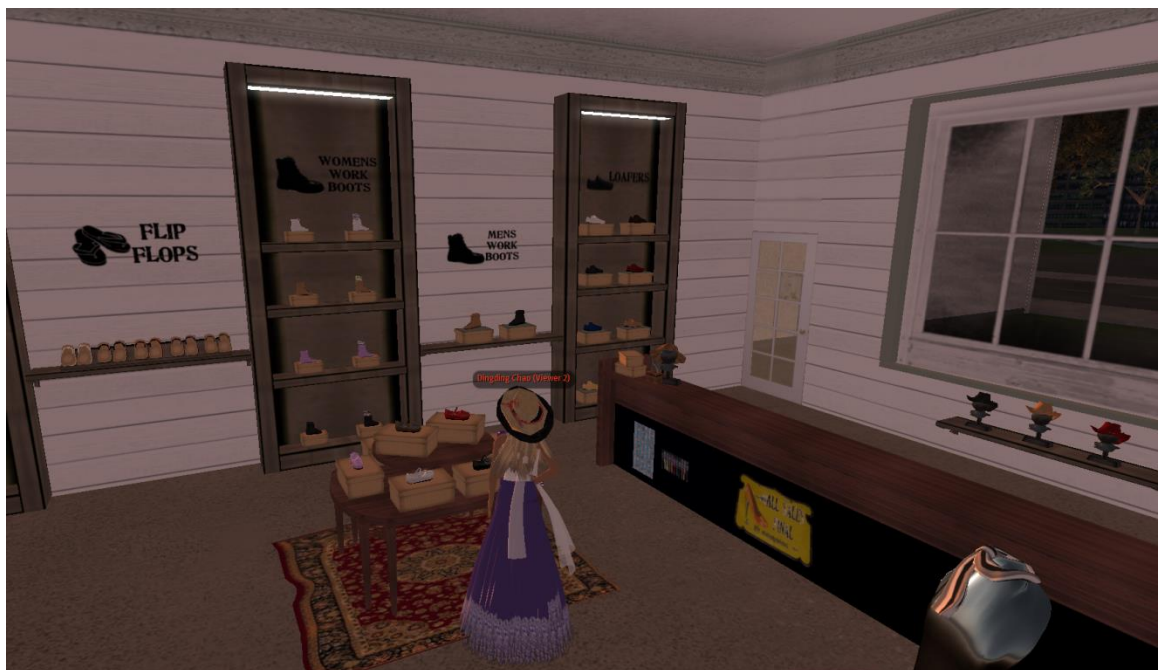

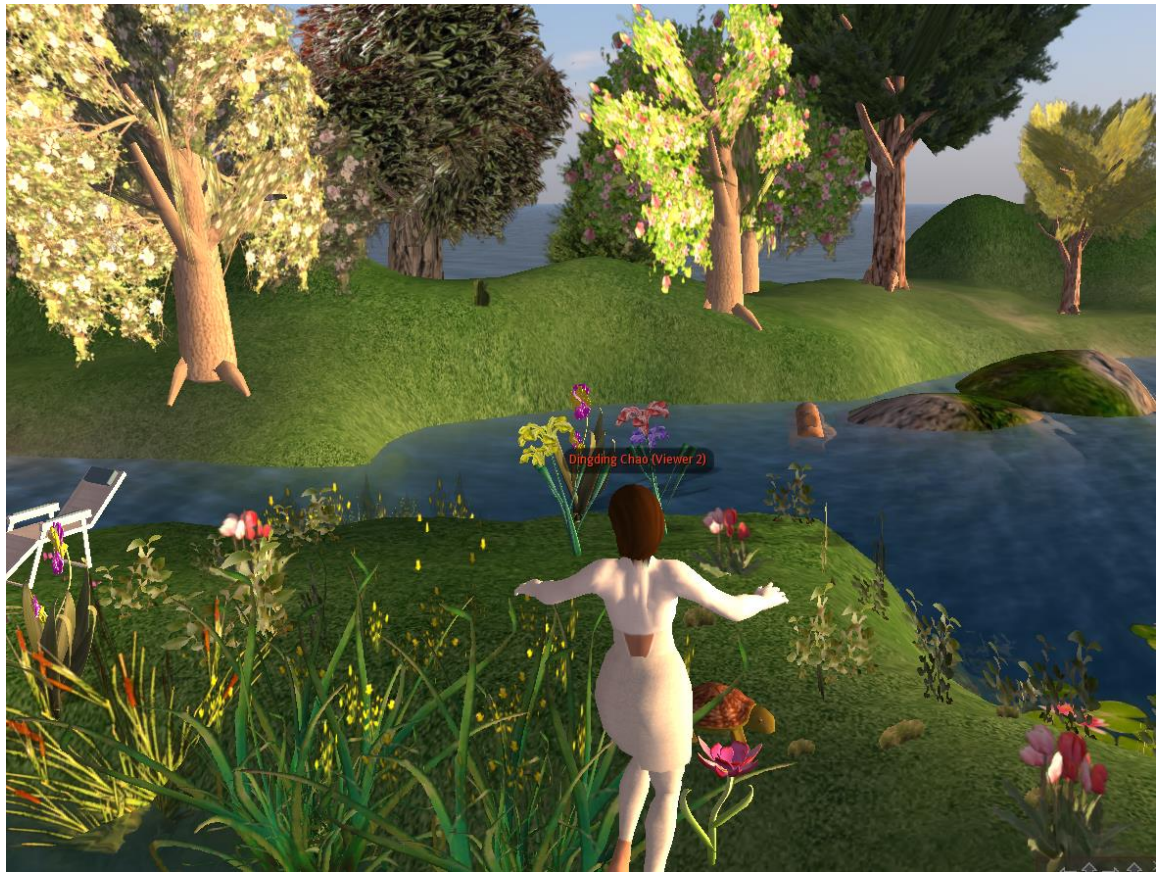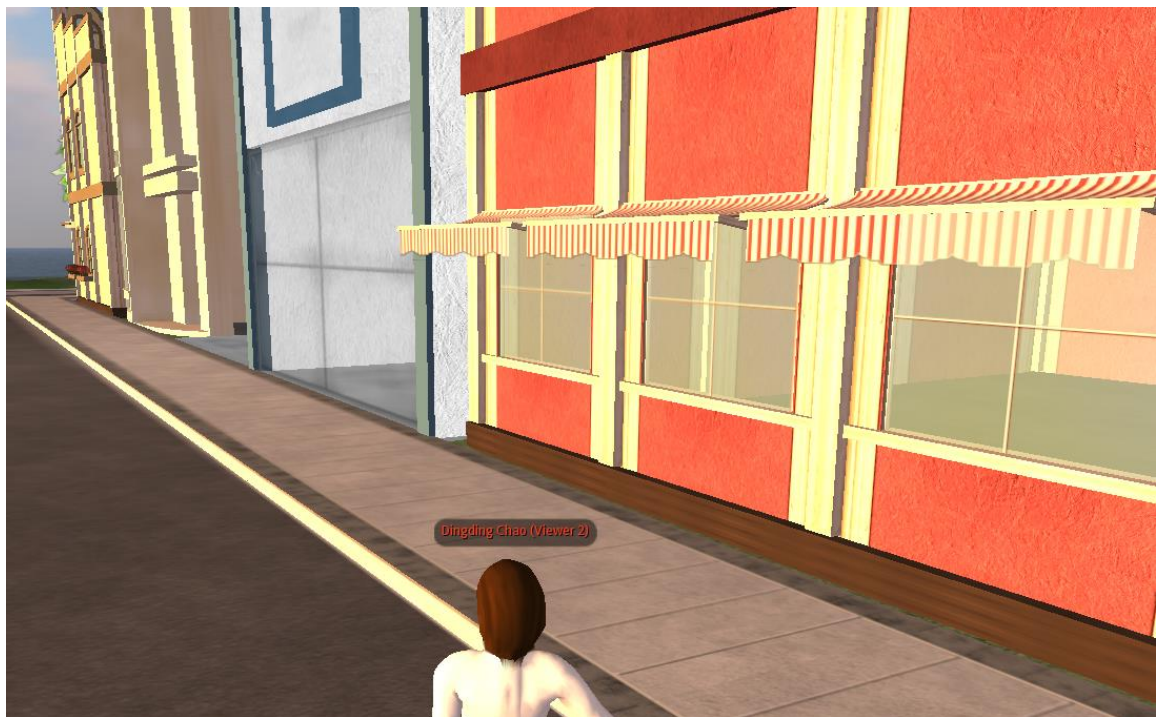

Supplement: Supplementary file 1 — Additional file 1: Appendix A. Screenshots used for Pre-examination. (PDF 2 MB) [file 40064_2013_1427_MOESM1_ESM.pdf]
